# The Impact of Infectious Diseases on Psychiatric Disorders: A Systematic Review

**DOI:** 10.7759/cureus.66323

**Published:** 2024-08-06

**Authors:** Okelue E Okobi, Oluwatoyin Ayo-Farai, My Tran, Chidalu Ibeneme, Cosmas O Ihezie, Oboatarhe B Ezie, Tinuade O Adeakin-Dada

**Affiliations:** 1 Family Medicine, Larkin Community Hospital, Miami, USA; 2 Family Medicine, Medficient Health Systems, Laurel, USA; 3 Family Medicine, Lakeside Medical Center, Belle Glade, USA; 4 Epidemiology and Public Health, Jiann-Ping Hsu College of Public Health, Georgia Southern University, Statesboro, USA; 5 Internal Medicine, Baptist Health, University of Arkansas for Medical Sciences, North Little Rock, USA; 6 Public Health, University of Toledo, Toledo, USA; 7 Orthopedics, Federal Teaching Hospital, Owerri, NGA; 8 Family Medicine, University of Benin, Benin, NGA; 9 Community and Family Medicine, Windsor University School of Medicine, Cayon, KNA

**Keywords:** immuno-psychiatric interface, cns infection, neuroinflammation, psychiatric disorders, infectious diseases

## Abstract

The complex connection between some infectious illnesses and some psychiatric disorders is an important area of study, with infections known to cause a diverse range of psychiatric symptoms. This association poses significant challenges for physicians in differentiating between mental illnesses induced by infections and those stemming from underlying psychiatric conditions. This study systematically synthesizes literature from various databases that explain the relationship between certain infectious diseases and specific psychiatric disorders. The systematic review explores mechanisms such as neuroinflammation, direct central nervous system (CNS) infection, and the interaction between the immune system and psychiatric conditions. The study examines various infectious pathogens, including viruses, bacteria, parasites, prion diseases, and fungi. An analysis of these findings is presented in the study's discussion section, along with a review of therapeutic methods such as drug use and psychological treatment. The review emphasizes the need for multidisciplinary teamwork and thorough clinical examinations in managing psychiatric symptoms caused by infections. It also highlights the significant role of public health measures in mitigating the impact of psychiatric diseases related to infectious illnesses. The study finds that current therapeutic methods include pharmacological and psychological treatments, which can effectively manage these conditions. The study has concluded that psychiatric manifestations are prevalent across various infectious diseases, including those caused by viruses, bacteria, parasites, and fungi. Key mechanisms identified include neuroinflammation, direct infection of the CNS, and the immuno-psychiatric interface, all of which contribute to the development of psychiatric symptoms. The future of managing these complex conditions lies in a comprehensive approach that combines clinical, therapeutic, and public health strategies.

## Introduction and background

The complex correlation between infectious diseases and psychiatric disorders highlights the need to comprehend the psychiatric consequences of infectious microorganisms. Infections may worsen or trigger psychiatric disorders, resulting in severe morbidity and mortality [1]. While the link between infectious diseases and mental health disorders has been known for millennia, the underlying processes remain unresolved. Infectious diseases have been associated with a range of psychiatric symptoms, including mood disorders, cognitive impairments, and neurocognitive issues [2]. Furthermore, the bidirectional correlations between infectious diseases and psychiatric disorders add complexity to clinical management, as psychiatric symptoms may both result from and contribute to the risk of developing infectious diseases [1,2]. For instance, studies have disclosed that approximately 25% to 40% of individuals with untreated syphilis infection might in the end develop tertiary disease, including psychiatric disorder, even though this might take up to 30 years after the infection [1-6]. Moreover, it has been shown that hospitalization due to infections considerably increased the risk of the patient developing later mood disorder by 63%, and hospitalization as a result of the autoimmune disorder increased it considerably by 45% [1-7].

The aim of this systematic review is to offer an in-depth analysis of infectious diseases' influence on mental health and identify gaps in current knowledge. Understanding the processes behind infection-induced psychological symptoms is critical for early diagnosis and effective medical treatment [3]. Moreover, determining the routes via which infectious pathogens impact mental health outcomes might guide the development of tailored treatment options to lessen the burden of psychiatric illness associated with infectious diseases [3]. This systematic review seeks insights into the complex relationship between infectious diseases and psychiatric disorders, eventually improving clinical practice and directing future research initiatives in this vital aspect of the relationship. 

## Review

Materials and methods

For this systematic review, the literature search was carried out on different online databases, including American Journal of Psychiatry, PubMed, BMC, Frontiers, Molecular Psychiatry, JAMA, Oxford Academic, MDPI, Elsevier, and Translational Psychiatry, for studies and literature on correlations between infectious illnesses and psychiatric disorders. The search employed Boolean operators with each feasible combination of Medical Subject Headings (MeSH) terms that included infectious diseases, psychiatric disorders, neuroinflammation, central nervous system (CNS) infection, and immuno-psychiatric interface. The search strategy involved two stages and was based on PRISMA guidelines for article selection and inclusion for systematic reviews. The first stage involved the independent screening of every retrieved article's title and abstract by two researchers. In instances of adequate data in the article’s abstract to inform the article retention or exclusion decision, the exclusion of the articles was made after full-text screening [4]. However, if not, articles with pertinent titles to this systematic review but having insufficient abstract data were additionally included for the second stage involving full-text screening [5]. The subsequent stage involved the full-text screening of retained articles for the inclusion and exclusion criteria. All instances of disputes were resolved by a third researcher who was required to decide on the suitability of the article for inclusion or exclusion, and this was arrived at through consultation and consensus. 

Inclusion and exclusion criteria

The inclusion criteria for this systematic review entailed original studies, including crossover design studies, randomized controlled trials, and prospective cohort studies that satisfied the following set criteria: studies on correlations between infectious illnesses and psychiatric disorders, English language published, and studies published within the last 10 years [4,5]. The excluded studies included sponsored clinical trials, opinion pieces, editorials, and narrative reviews. The articles' abstract evaluation led to the removal of 350 articles. In addition, the extraction of important information from the identified and included eligible studies was done based on the following: (a) general characteristics of the study, including the names of the authors, study year, year of publication, and employed sampling methods; (b) characteristics of the study population, including the sample size, gender, race, and age, as well as follow-up; (c) type of intervention and duration; and (d) key findings of the study. The summary of the findings of the studies included in this systematic review has been presented in Table 1.

**Table 1:** A summary of the findings of the studies and articles included in this systematic review PPE, personal protective equipment; HCV, hepatitis C virus; NCDs, non-communicable diseases

Author names	Study title	Publication year	Summary of findings
McGinty E, Baller J, Azrin S, Juliano-Bult D, Daumit G	Quality of medical care for persons with serious mental illness: A comprehensive review.	2015	The study disclosed that persons with severe mental illnesses often receive poor quality healthcare services in comparison to the general population. This also includes lower rates of preventive care, screenings, and chronic condition management.
DeKock JH, Latham HA, Leslie SJ, et al.	A rapid review of the impact of COVID-19 on the mental health of healthcare workers: Implications for supporting psychological well-being.	2021	The study disclosed that increased levels of anxiety, depression, distress, and insomnia existed among healthcare workers, with the various risk factors contributing to such psychological health issues including closer contact with COVID-19 patients, concerns regarding the safety of one’s family, underlying health conditions, and lack of PPE.
Thornton C, Chaisson LH, Bleasdale SC	Characteristics of pregnant women with syphilis and factors associated with congenital syphilis at a Chicago hospital.	2022	The study disclosed that a substantial proportion of the women diagnosed with syphilis were either inadequately treated or did not receive timely treatment. The major risk factors for congenital syphilis included insufficient and late prenatal care, substance abuse, and a sexually transmitted infections history. The study stressed the significance of early screening and treatment to prevent congenital syphilis, in addition to emphasizing the need for targeted interventions to tackle identified risk factors.
Bransfield R, Cook M, Bransfield D	Proposed Lyme disease guidelines and psychiatric illnesses.	2019	The study disclosed the probable consequences of not recognizing the psychiatric manifestations of Lyme disease, including an increased risk of suicide, substance abuse, and violence. The study also highlighted the need for significant revisions to the guidelines, stressing the need for increasingly accurate diagnostic criteria and recognition of the severe mental health implications of Lyme disease
Bransfield R	Neuropsychiatric Lyme borreliosis: An overview with a focus on a specialty psychiatrist’s clinical practice.	2018	The study disclosed that Lyme disease may result in a wider range of mental health issues, including anxiety, depression, bipolar disorder, and cognitive impairments. Such symptoms normally present alongside other physical complaints that may be exacerbated by different co-infections.
Abo-Al-Ela HG	Toxoplasmosis and psychiatric and neurological disorders: A step toward understanding parasite pathogenesis.	2020	The research disclosed how the *Toxoplasma gondii* parasite contributes to the pathogenesis of conditions that include schizophrenia, bipolar disorder, and neurodegenerative diseases. The study has further disclosed that *T. gondii* can alter neurotransmitter systems, immune responses, and brain function, potentially leading to behavioral and cognitive changes.
Adinolfi LE	Chronic hepatitis C virus infection and neurological and psychiatric disorders: An overview.	2015	The study explored the existing correlations between chronic HCV infection and an array of psychiatric and neurological disorders. It disclosed that a substantial percentage of individuals with chronic HCV tend to experience neuropsychiatric symptoms that include cognitive impairment, anxiety, depression, and fatigue. The conditions have been noted to result from the virus’ neurotoxic effects, immune-mediated mechanisms, and metabolic disturbances.
Serrano-Castro PJ, Estivill-Torrús G, Cabezudo-García P, et al.	Impact of SARS-CoV-2 infection on neurodegenerative and neuropsychiatric diseases: A delayed pandemic?	2020	The study disclosed that SARS-CoV-2 was a neuroinvasive virus capable of inducing a cytokine storm, which can have lasting effects on the central nervous system. Therefore, the researchers proposed that the virus may exacerbate or trigger the onset of neuro-inflammatory conditions, potentially leading to a delayed pandemic of neurodegenerative and neuropsychiatric disorders.
Klein RS, Garber C, Howard N	Infectious immunity in the central nervous system and brain function.	2017	The study has revealed how immune responses to infections may influence the CNS processes, potentially impacting brain function and behavior. The study also focused on the dual role of the immune system with regard to the protection of the brain from pathogens and contributing to neurological disorders when dysregulated.
De Picker LJ	The future of immunopsychiatry: Three milestones to clinical innovation.	2021	In outlining the emergence of the immune-psychiatry field, this study has highlighted three critical milestones required for the advancement of the field, including the definition of patient populations in the immune-psychiatric continuum, demonstration of clear clinical benefits, and integration of the findings with other biological psychiatry paradigms.
Bhaskar S, Bradley S, Israeli-Korn S, et al.	Chronic neurology in COVID-19 era: Clinical considerations and recommendations from the REPROGRAM consortium.	2020	In offering insights into the neurological complications linked to COVID-19, both chronic and acute, the study has disclosed that COVID-19 may result in various neurological issues, including Guillain-Barré syndrome, strokes, and encephalitis, among others.
Menzies RE, Menzies RG	Death anxiety in the time of COVID-19: Theoretical explanations and clinical implications.	2020	The study explored the effects of the COVID-19 pandemic on death anxiety, which is a type of existential distress regarding the fear of death. The study disclosed that the pandemic intensified this anxiety as a result of increased exposure to death-linked cues, including the daily mortality statistics and various public health interventions such as mask-wearing.
Palmer K, Monaco A, Kivipelto M, et al.	The potential long-term impact of the COVID-19 outbreak on patients with non-communicable diseases in Europe: Consequences for healthy aging.	2020	The study explored the potential long-term impacts of the COVID-19 disease on patients with NCDs in Europe in relation to the outcomes for healthy aging. The study has disclosed that disruptions in healthcare services, delays in diagnoses, and reduction in management of chronic conditions during the pandemic were linked to the exacerbation of NCDs, resulting in poorer health outcomes and increased mortality rates.
Frontera JA, Simon NM:	Bridging knowledge gaps in the diagnosis and management of neuropsychiatric sequelae of COVID-19.	2022	The study disclosed that there was a significant association between neuropsychiatric sequelae and COVID-19, resulting in cognitive impairment, anxiety, and mood disorders. The study has emphasized the need for the performance of comprehensive research to enable the understanding of mechanisms driving such conditions in addition to advocating for standardized diagnostic criteria and treatment protocols.

Data collection process

Extraction of data from included studies was carried out with the author’s monitoring. Potential disputes and discrepancies were resolved through consultations and discussions, and the information on the main author, year of publication, study location, sample size, rate of response, and screening tool utilized was extracted independently from each study by the author through the use of a standard data extraction format.

Quality assessment

The assessment of the included studies' quality in this systematic review was performed using the Joanna Briggs Institute quality assessment tool. Thus, each publication’s scoring was carried out through the use of the frequency scales with yes, no, unclear, and not applicable responses. The overall quality score of every study was then calculated on the basis of the overall amount of positive scores received.

Statistical analysis

For this systematic review, each statistical analysis was carried out using the v3 comprehensive meta-analysis software. The individual studies' prevalence rates were pooled using the random effects meta-analysis. The heterogeneity of the studies reviewed was evaluated using I2 statistics. The I2 statistics values, including 25%, 50%, and 75%, are representative of low, medium, and high heterogeneity, respectively. The attributes of the study, including research design, sample size, publication year, and study location were used to evaluate the possible heterogeneity sources. For each statistical analysis conducted in relation to statistical significance, the P-value was set at 0.05.

**Figure 1:**
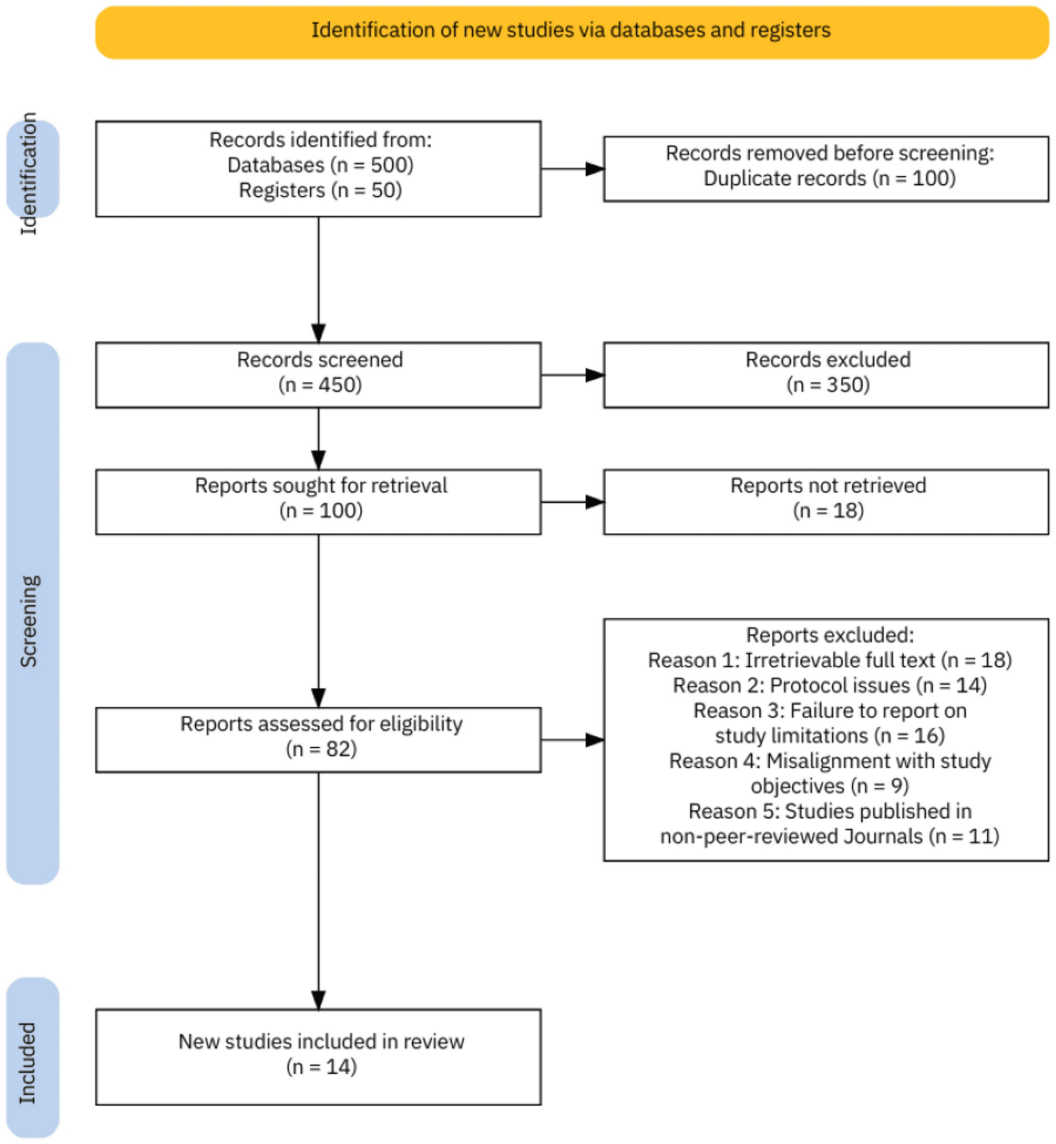
PRISMA flow diagram indicating the study/article selection process for this study n, number

A total of 550 references were retrieved via database searches and other sources. After removing 100 duplicates, 450 unique records remained for screening. During the title and abstract screening phase, 350 publications were removed because of their irrelevance to the research topic. Subsequently, several reviewers independently examined 100 possibly relevant full-text articles according to preset selection criteria. Following the full-text assessment, 68 papers were discarded for various reasons, ranging from (i) irretrievable full text (18 articles), (ii) protocol issues (14 articles), (iii) failure to report on study limitations (16 articles), (iv) misalignment with the study objectives (nine articles), and (v) studies published in non-peer-reviewed journals (11 articles). In the end, 31 studies met the inclusion criteria and were included in the qualitative synthesis. Figure 1 below provides a detailed overview of the literature search and selection process conducted using PRISMA.

Discussion

Viral Infections

Human immunodeficiency virus (HIV): Research has indicated a substantial prevalence of psychiatric disorders among patients with HIV infection. Studies have identified mental symptoms, including sadness, anxiety, and cognitive impairment, which are ascribed to both neuroinflammation and the direct invasion of the CNS by the virus [6]. The presence of HIV in the CNS may lead to neurocognitive abnormalities, including HIV-associated neurocognitive disorders (HAND), further worsening mental morbidity in infected patients. In addition, the chronic inflammatory response caused by HIV infection may contribute to the development and progression of mental symptoms, requiring comprehensive therapy techniques to address both viral and psychiatric elements of the illness [7,8].

COVID-19: Emerging research reveals a strong link between COVID-19 and mental symptoms, including anxiety, despair, and post-traumatic stress disorder (PTSD). The psychological effect of the pandemic goes beyond acute infection, with long-term mental health consequences identified in survivors having persistent symptoms such as tiredness, cognitive impairment, and mood abnormalities [9-11]. The multifaceted etiology of mental symptoms in COVID-19 includes complex interactions between the direct effects of the virus on the CNS, systemic inflammation, psychosocial stresses, and the indirect implications of isolation and quarantine procedures [9-11]. Addressing the psychiatric sequelae of COVID-19 is crucial for establishing timely treatments and support services to decrease the long-term load on mental health systems and enhance overall patient outcomes.

Bacterial Infections

Syphilis: Neurosyphilis, a late-stage manifestation of Treponema pallidum infection, may present diverse neuropsychiatric symptoms, including psychosis, cognitive impairment, and mood problems [12,13]. The various clinical symptoms of neurosyphilis underline the significance of early detection and treatment to avoid irreversible neurological and mental consequences. Psychiatric symptoms may precede or coexist with other neurological indications, providing diagnostic complications for doctors. The reemergence of syphilis in certain groups highlights the enduring significance of this ancient bacterium as a potential cause of psychiatric disorders in the modern era [12,13].

Lyme disease: Lyme disease, caused by the spirochete Borrelia burgdorferi, is linked with a variety of mental symptoms, including mood disorders, cognitive impairment, and psychosis [14,15]. However, the persistent issues regarding the diagnosis and treatment of Lyme disease-related mental symptoms continue to pose significant challenges, leading to diagnostic ambiguity and heterogeneity in clinical practice. The complex interaction between host immunological response, neuroinflammation, and direct neurotoxic effects of the spirochete highlights the need for immediate actions and additional study to clarify the pathophysiological processes underlying Lyme disease-associated psychiatric disorders [14,15]. Improved diagnostic techniques and evidence-based treatment options are urgently needed for successfully treating mental symptoms in individuals with Lyme disease and limiting long-term impairment and morbidity [14,15]. 

Parasitic and Fungal Infections

Toxoplasmosis: *Toxoplasma gondii* infection has been linked to the pathophysiology of schizophrenia, with studies indicating a relationship between seropositivity for *T. gondii *antibodies and an increased risk of developing schizophrenia [16,17]. The neurotropic features of *T. gondii *allow the parasite to influence host neurochemistry and immunological response, possibly leading to the development of psychiatric disorders. Although the specific processes underpinning the link between toxoplasmosis and schizophrenia remain unclear, the accumulating evidence implies a complex interaction between host genetics, immunological dysregulation, and external factors in disease susceptibility and development [16,17]. Further study is necessary to unravel the role of *T. gondii *infection in the pathophysiology of schizophrenia. This research could lead to the discovery of innovative treatment strategies, offering hope for decreasing mental morbidity in afflicted patients.

Cryptococcal meningitis: Immunocompromised persons, especially those who have a severe HIV infection or other manifestations of cellular immunodeficiency, are at higher risk of acquiring cryptococcal meningitis, a life-threatening fungus infection of the CNS caused by *Cryptococcus neoformans* [16,17]. Psychiatric signs of cryptococcal meningitis can include unstable mental state, hallucinations, delusions, and personality abnormalities, reflecting the wide range of CNS involvement reported in afflicted individuals. Early identification and care of cryptococcal meningitis are critical to minimize neurological complications and improve clinical outcomes. Nevertheless, difficulties associated with diagnosing CNS fungal infections, especially in resource-limited settings, emphasize the significance of raising awareness among healthcare providers concerning the psychiatric presentations of these diseases and establishing targeted screening and diagnostic algorithms to promote timely intervention and maximize patient care. The key studies that focused on the infectious diseases and psychiatric disorders selected for this study have been summarized in Table 2 [6-21]. 

**Table 2:** Summary of key studies on infectious diseases and psychiatric disorders HIV, human immunodeficiency virus

Infectious disease	Study type	Key findings	Implications
HIV	Observational	High prevalence of depression, anxiety, and cognitive impairment linked to neuroinflammation and CNS invasion.	Comprehensive treatment strategies are needed to address both viral and psychiatric aspects [6,8].
COVID-19	Observational	Anxiety, depression, PTSD in survivors, long-term mental health effects due to neuroinflammation, and psychosocial stress.	It is important to have timely interventions and support services to mitigate long-term mental health burdens [9-10].
Syphilis (neurosyphilis)	Case reports	Neuropsychiatric symptoms like psychosis and cognitive impairment emphasize early detection and treatment.	Early diagnosis and treatment are critical to prevent irreversible neurological and psychiatric outcomes [11-12].
Lyme disease	Review	Associated with mood disorders, cognitive impairment, and psychosis, highlighting diagnostic challenges.	Enhanced diagnostic and treatment approaches are necessary to limit long-term impairment [14-15].
Toxoplasmosis	Review	The link between *T. gondii* infection and schizophrenia, with neurotrophic effects influencing host neurochemistry.	Further research is required to understand the pathophysiology and develop innovative treatment strategies [16-17].
Cryptococcal meningitis	Observational	Immunocompromised individuals at risk of cryptococcal meningitis. Psychiatric symptoms include unstable mental state, hallucinations, and delusions.	Awareness among healthcare providers and targeted screening algorithms for timely intervention [20-21].

Mechanisms linking infectious diseases and psychiatric disorders

Neuroinflammation

Neuroinflammation modulates the bidirectional link between infectious illnesses and psychiatric disorders. Inflammatory cytokines, including tumor necrosis factor-alpha (TNF-α) and interleukin-6 (IL-6), are involved in the pathophysiology of psychiatric symptoms via altering neurotransmitter systems, synaptic plasticity, and neurogenesis [18,19]. Additionally, dysregulated immune responses inside the CNS may breach the blood-brain barrier, enabling the influx of peripheral immune cells and pro-inflammatory mediators into the brain parenchyma [18,19]. Therefore, neuroinflammation leads to neuronal dysfunction, synaptic pruning, and neurodegeneration, ultimately increasing psychiatric morbidity in persons with infectious disorders [18,19]. Recognizing the molecular pathways behind neuroinflammation is critical for creating tailored treatment approaches to reduce psychiatric symptoms and improve patient outcomes.

Direct CNS Infection

Infectious diseases may enter the CNS via different mechanisms, including hematogenous spread, retrograde neuronal transmission, and direct injection [20,21]. Case studies have reported cases of direct brain infection leading to mental symptoms such as psychosis, mood disorders, and cognitive impairment [20,21]. The neurotropic features of some viruses allow them to elude host immune systems and develop persistent infection inside the CNS, resulting in chronic neuroinflammation and neuronal death [20,21]. The clinical symptoms of CNS illness differ based on the site and level of neural involvement, reflecting the range of psychiatric presentations encountered in infected patients [20,21]. Recognizing the exact pathogens and pathophysiological pathways behind CNS illness is critical for directing focused antimicrobial treatment and minimizing long-term neurological sequelae.

Immuno-Psychiatric Interface

The complex relationship between the immune system and the CNS is the key factor in the etiology of psychiatric disorders in the context of infectious diseases. Dysregulated immunological responses, characterized by aberrant cytokine production, microglial activation, and T-cell infiltration, can trigger psychiatric symptoms through neurotoxic effects on synaptic function and neurotransmitter metabolism [22]. Chronic immunological activation, often associated with chronic infection or systemic inflammation, significantly exacerbates mental morbidity by perpetuating neuroinflammatory processes and impairing neuroplasticity [22]. Moreover, the bidirectional communication between the immune and neuroendocrine systems plays a crucial role in defining the clinical course of psychiatric disorders, influencing stress responsivity, mood control, and cognitive function in patients with infectious diseases [22]. This highlights the need for a comprehensive understanding of the relationships between immunological dysregulation and psychiatric disease, as it is essential for developing targeted immunomodulatory medicines and tailored interventions that aim to restore CNS homeostasis and improve mental health outcomes.

Clinical implications and management

**Figure 2:**
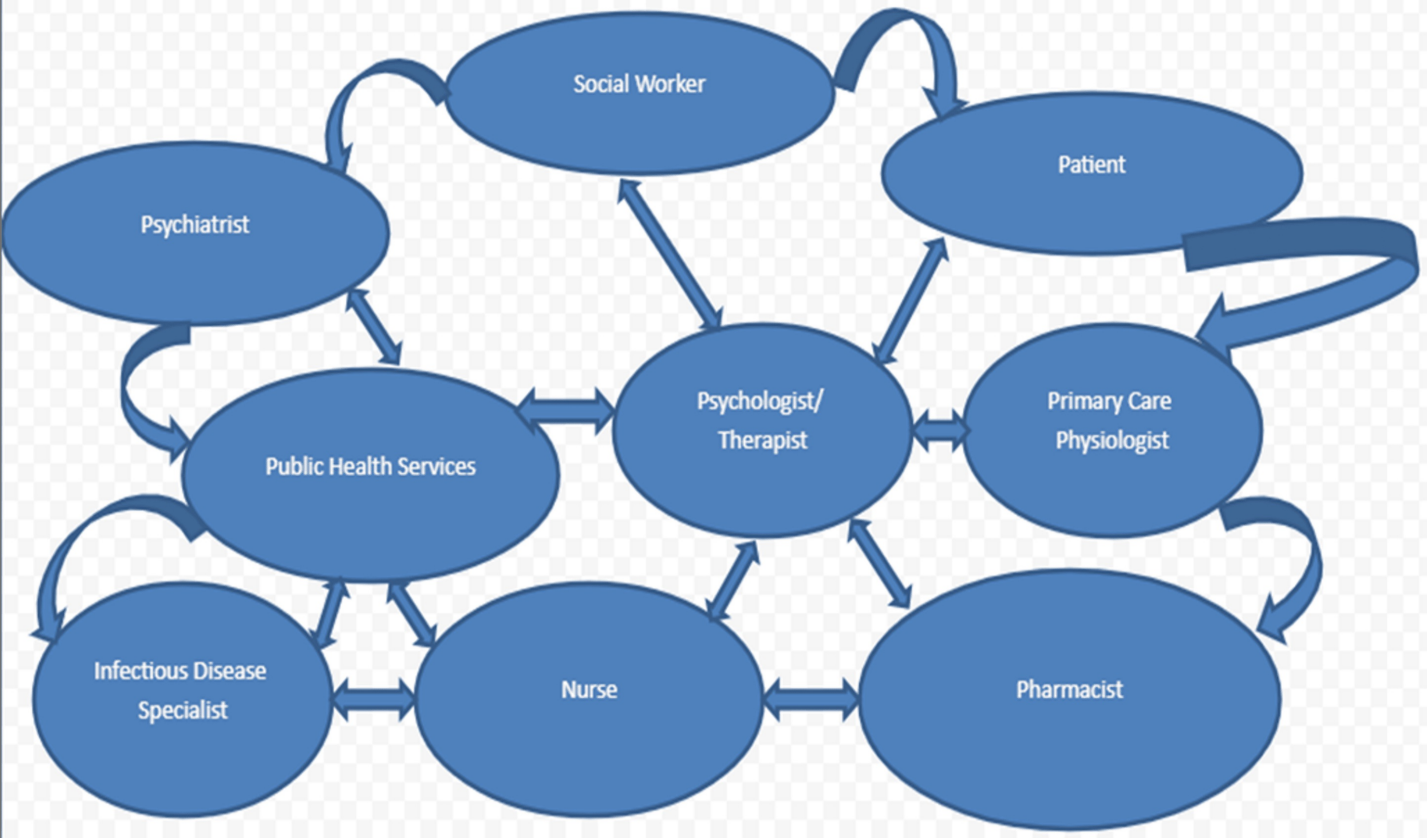
Integrated care model for clinical management

Distinguishing between primary psychiatric disorders and infection-induced psychiatric symptoms is a challenging task due to overlapping clinical presentations and common pathophysiological pathways. The psychiatric symptoms of infectious diseases can resemble mood disorders, psychotic disorders, or cognitive impairments, complicating the differential diagnosis [23,24]. Moreover, the varying start and course of mental symptoms in the context of acute or chronic illnesses further complicate the diagnostic process [23,24]. However, the value of interdisciplinary teamwork between infectious disease specialists, psychiatrists, neurologists, and clinical psychologists cannot be overstated. This collaboration is crucial to effectively identify and treat infection-related mental problems [23,24]. Thorough clinical evaluations, including extensive medical history, physical examination, laboratory tests, and neuroimaging studies, are necessary to identify the root of the problem and direct effective treatment approaches [23,24]. The integrated care model for clinical management has been provided in Figure 2.

Treatment approaches

Pharmacological Interventions

Treatment of infection-induced psychiatric symptoms frequently needs a multifaceted strategy addressing both the infectious agent and its associated neuropsychiatric disease. Pharmacotherapy has a vital role in addressing acute psychiatric symptoms associated with infectious illnesses [25-27]. Antipsychotic medications may be used to treat psychosis, whereas antidepressants and anxiolytics help improve mood abnormalities and anxiety symptoms [25-27]. Concurrent antimicrobial treatment targeting the causal organism is critical for eliminating infection and limiting illness development. However, choosing suitable psychotropic and antimicrobial medications calls for careful evaluation of potential drug interactions, side effects, and specific patient characteristics to enhance therapy effectiveness and minimize adverse outcomes [25-27]. 

Psychotherapy Interventions

In addition to pharmacological therapies, psychotherapy modalities, such as cognitive-behavioral therapy (CBT), supportive therapy, and psychoeducation, are critical components of comprehensive treatment methods for infection-induced mental illnesses [25-27]. Psychotherapy seeks to address maladaptive thinking patterns, coping strategies, and psychosocial stresses leading to psychiatric symptomatology while promoting resilience and adaptive coping mechanisms [25-27]. Integrating psychotherapy into the treatment plan boosts patient participation, promotes emotional control, and enables long-term recovery from mental illness.

Strengths and limitations of this study

The notable strengths of the present systematic review include the observation that it has utilized an effective methodology that has enabled the identification of appropriate and high-quality articles for inclusion in the study. Additionally, the other strength of this study includes the observation that the studies included are drawn from various regions of the globe, which makes the findings of this systematic review increasingly generalizable to different populations. Nevertheless, a number of limitations have also been noted including the observation that a number of analyses in the studies reviewer were of associational nature, which makes it impossible to effectively draw definitive conclusions regarding causality. Moreover, the correlations between infectious diseases and certain psychiatric disorders including mood disorders are prone to be influenced by numerous confounding and interacting variables that were not evaluated in this systematic review, including the variations in the immune-related genes’ allele frequencies, social support levels, social capital, and the prescription patterns of antibiotics.

Future directions and research gaps

The ongoing emergence of new infectious diseases, such as coronaviruses, influenza viruses, and multidrug-resistant bacteria, underscores the need to understand their potential psychological impact and develop appropriate preventive and control strategies [28]. Future study initiatives should clarify the neurobiological processes that underlie infection-induced psychiatric symptoms, longitudinal studies to evaluate chronic psychiatric outcomes, and targeted therapies suited to the distinct pathophysiological profiles of emerging infectious diseases [28]. Furthermore, coordinated efforts between academics, doctors, public health organizations, and politicians are vital for minimizing the worldwide impact of infectious illnesses on mental health and well-being.

Advancements in precision medicine, spanning genetic profiling, biomarker identification, and tailored therapy techniques show promise for increasing the accuracy of psychiatric diagnosis and maximizing therapeutic results in persons with infection-induced psychiatric disease [29,30]. The integration of genetic and biomarker investigations into clinical practice helps discover susceptibility genes, prediction markers, and treatment response biomarkers linked with infection-related mental illness [29,30]. Tailoring treatment plans according to individual genetic profiles and molecular signatures allows targeted treatments to deal with the primary pathophysiological mechanisms driving psychiatric symptoms, thus improving the effectiveness of treatment, reducing negative consequences, and ultimately boosting patient outcomes [29,30].

Effective treatment of infection-induced psychological disorders demands a comprehensive public health strategy involving prevention, early intervention, and integrated healthcare delivery systems. Public health policies targeting infectious illnesses, such as vaccination campaigns, infection control measures, and public health education programs, are crucial in lowering the incidence and prevalence of infection-related mental morbidity [31]. Early identification and timely care are essential for reducing the influence of infection-induced psychiatric symptoms on personal and population-level mental health outcomes [31]. Also, encouraging cooperation among healthcare providers, community organizations, and governmental agencies promotes the creation and implementation of integrated care models focusing on the complex link between infectious diseases and psychiatric disorders, which improves access to timely and extensive mental healthcare services [31].

## Conclusions

This comprehensive review highlights the intricate connection between some infectious diseases and some psychiatric disorders. The findings reveal the prevalence of psychiatric manifestations across various infectious diseases, including those caused by viruses, bacteria, parasites, and fungi. Key mechanisms identified include neuroinflammation, direct infection of the CNS, and the immuno-psychiatric interface, all of which contribute to the development of psychiatric symptoms. Also, the review highlights the critical need for heightened awareness among healthcare professionals regarding the psychiatric implications of infectious diseases. Public health measures focused on early detection, prevention, and comprehensive treatment of infectious diseases can substantially reduce the psychiatric morbidity associated with these conditions. Future research should aim to deepen our understanding of the complex interactions between infectious pathogens and psychiatric disorders. Longitudinal studies are essential to elucidate the long-term psychiatric outcomes of infectious diseases. Precision medicine approaches, including genetic and biomarker research, hold promise for identifying individual susceptibilities to infection-induced psychiatric conditions. Multidisciplinary collaboration remains vital in optimizing patient care and outcomes, emphasizing the need for integrated strategies in managing the dual burden of infectious diseases and psychiatric disorders.
